# Stabilizing the retromer complex rescues synaptic dysfunction and endosomal trafficking deficits in an Alzheimer’s disease mouse model

**DOI:** 10.1186/s40478-025-02096-8

**Published:** 2025-09-10

**Authors:** David Ramonet, Anna Daerr, Martin Hallbeck

**Affiliations:** Department of Biomedical and Clinical Sciences and Department of Clinical Pathology, Linköping University, 58185 Linköping, Sweden

## Abstract

Disruptions in synaptic transmission and plasticity are early hallmarks of Alzheimer’s disease (AD). Endosomal trafficking, mediated by the retromer complex, is essential for intracellular protein sorting, including the regulation of amyloid precursor protein (APP) processing. The VPS35 subunit, a key cargo-recognition component of the retromer, has been implicated in neurodegenerative diseases, with mutations such as L625P linked to early-onset AD. Despite growing evidence for retromer dysfunction in AD, its role in synaptic pathology and neuroinflammation remains incompletely understood. Here, we investigate the acute molecular effects of retromer stabilization in the 5xFAD mouse model of AD using the pharmacological chaperones R55 and R33, previously identified to enhance VPS35 stability. Following intracranial stereotaxic injections, we performed transcriptomic profiling, quantitative histology, and immunohistochemistry to assess synaptic function, neuroinflammation, and endosomal trafficking. Our findings reveal that retromer stabilization reverses multiple AD-associated molecular changes. R55 treatment significantly reduced Aβ-related pathology, normalized synaptic gene expression, and restored long-term potentiation (LTP)-associated pathways, including Gria1 (AMPA receptors), Grip1, and semaphorin/plexin signaling. Additionally, retromer stabilization counteracted dysregulated calcium signaling by modulating Ryr2 and L-type calcium channel expression. Beyond synaptic effects, we observed broad transcriptional and structural changes in the endosomal system. Notably, R55 treatment decreased VPS13 family gene expression, implicated in membrane contact site regulation, while increasing RAB7 levels, suggesting enhanced late-endosomal recycling. VPS35-positive vesicles were redistributed away from the nucleus, indicating restored intracellular trafficking dynamics. In the neuroinflammatory domain, retromer stabilization modulated microglial activation, shifting towards a profile characterized by balanced pro-inflammatory (Il1, Nfkb2) and anti-inflammatory (Il4r, Il13ra1, Stat6) markers, consistent with disease-associated microglia (DAM) phenotypes. Together, these findings demonstrate that retromer dysfunction contributes to key AD pathologies, including synaptic dysfunction and neuroinflammation, and that pharmacological retromer stabilization can restore cellular homeostasis. Given that 5xFAD mice lack direct VPS35 mutations, our results suggest that retromer-targeting strategies may be applicable to both familial and sporadic AD, offering a promising therapeutic avenue for modifying disease progression.

**Supplementary Information:**

The online version contains supplementary material available at 10.1186/s40478-025-02096-8.

## Introduction

Synaptic dysfunction is an early hallmark of Alzheimer's disease (AD) and other neurodegenerative disorders, driven by disruptions in synaptic transmission and plasticity. In AD, beta-amyloid (Aβ) accumulates within synapses, impairing neurotransmitter release, receptor signalling, and dendritic spine stability, strongly implicating Aβ in synaptic pathology [7, 28]. However, the mechanisms underlying synaptic Aβ accumulation remain incompletely understood, and effective intervention strategies are lacking.

The endolysosomal pathway is crucial for intracellular trafficking including cell membrane vesicle transport and recycling [31]. Clathrin-mediated endocytosis initiates the formation of early endosomes, which mature into multivesicular bodies and are subsequently routed either to the Golgi apparatus via the retrograde pathway or recycled to the plasma membrane. The retromer complex is critical in this process because it determines cargo selection for endosomal transport. The retromer complex plays a crucial role in this process by selecting cargo for endosomal transport, including neuronal receptors, but also amyloid precursor protein (APP) and APP-processing enzymes. Given the rapid turnover of synaptic receptors via endocytosis, any disruption in cargo selection can significantly impact synaptic function [13]. Moreover, the endosomal system is integral to protein maturation, as proteins critical for synaptic activity, including APP, undergo proteolytic processing *en route* from the Golgi to the plasma membrane [34, 37, 39, 43].

A key component of the retromer complex is VPS35, which regulates its assembly, stability, and cargo recognition [6]. Mutations in VPS35, such as D620N, are linked to autosomal dominant Parkinson’s disease (PARK17) [42, 46], while the L625P variant has been associated with early-onset AD [34]. The retromer complex influences APP processing by directing its trafficking within the endosomal system, and alterations in this pathway precede Aβ deposition in AD [9]. Notably, VPS35 expression declines early in the entorhinal cortex, mirroring the spatiotemporal progression of AD pathology [36]. Thus, in AD, early dysfunction in the endosomal and retromer system coincide with the synaptic dysfunctions. VPS35 dysfunction leads to APP accumulation in early endosomes, increased Aβ production and exosomal release, and enlarged axonal endosomes [9, 16, 20]. Furthermore, Aβ itself downregulates VPS35 protein levels, with Aβ colocalizing with VPS35-positive endosomes [1]. Interestingly, the retromer complex also plays a role in microglial function, influencing microglial activation states that are critical in AD pathogenesis [33].

Given the pivotal role of VPS35, efforts have been made to pharmacologically stabilize the retromer complex. A small-molecule screening identified two thiopenthene thiourea compounds, R55 and R33, as pharmacological chaperones that enhance the stability of the binding of VPS35-VPS29 [26]. In vitro, these compounds increased VPS35 and VPS26 protein levels in mouse primary cortical neurons and reduced APP processing, lowering endogenous Aβ42 and Aβ40 levels at low micromolar concentrations (5 µM) [26]. It is now believed that the effect of R55 and R33 is primarily by restoring the function and localization of the retromer complex [10]. However, despite these promising results, there is limited understanding of the therapeutic potential of retromer stabilization in in vivo AD models, exacerbated by the retraction of several key publications in the field [22, 41]. Furthermore, while in vitro data suggest Aβ involvement, the broader molecular mechanisms underpinning retromer-mediated neuroprotection remain unclear. Given that VPS35 interacts with multiple key players in AD pathophysiology, its dysfunction may serve as a central failure hub in disease progression.

In this study, we investigate the acute molecular effects of retromer stabilization in the 5xFAD/C57BL6 murine AD model following 24-h intraparenchymal injections of R55 and R33. Our goal is to map the downstream signalling pathways affected by retromer stabilization and assess the potential to reverse AD-associated molecular events. Given the limited pharmacokinetic data on these compounds and the challenges of long-term intraparenchymal delivery, we focus on the immediate biochemical responses to VPS35 stabilization.

## Materials and methods

### Drugs

The thiophene thioureas, R55 and R33, were obtained as hydrochloride salts from CaymanChem (Ann Arbor, MI) and MedKoo (Morrisville, NC), respectively. To prevent degradation due to hydrolysis and oxidation, the compounds were dissolved in high-purity anhydrous DMSO at a concentration of 300 mM, aliquoted, and stored at − 70 °C. Immediately before intracranial microinjection, one aliquot was thawed and diluted to 10 mM under sterile conditions using freshly prepared 20% sulfobutylether beta-cyclodextrin (Thermo Fisher) in saline. A vehicle control solution containing the same proportions of DMSO and beta-cyclodextrin (final concentration of 3% DMSO) was prepared alongside the drug solutions to ensure consistency across treatments.

Although pharmacokinetic data for R55 and R33 is not available, the estimated final cortical concentration following a 500 nL injection was approximately 25 µM, assuming homogeneous distribution. This concentration exceeds the estimated dissociation constant (Kd) for VPS35 stabilization (~ 5 µM) and the IC₅₀ for Aβ reduction (~ 12 µM) in primary neurons, as previously described (Mecozzi et al., 2014).

### Animals and surgeries

Experiments were conducted using 16-week-old female 5xFAD/C57BL6 mice (B6), specifically Cg-Tg(APPSwFlLon, PSEN1*M146L*L286V)6799Vas/Mmjax, obtained from The Jackson Laboratory (JAX) and locally bred with C57BL/6 J mice from the same source. Compounds were administered via stereotaxic microinjection into the lateral parietal associative cortex using the following coordinates: caudal − 2.06 mm, lateral −1.5 mm, and ventral − 0.8 mm. Infusions were performed at a rate of 100 nL/min, with a total volume of 500 nL.

Twenty-four hours post-injection, mice were euthanized, and brains were rapidly dissected. For the transcriptomics study, six mice per treatment group (n = 6 per group) were used. A cortical tissue block measuring approximately 1 mm3 was isolated from each hemisphere, with the injection site serving as the focal point in the ipsilateral hemisphere. The contralateral hemisphere was used as an internal control. Care was taken during microdissection to ensure that no parts of the corpus callosum were included, thereby preventing contamination from interhemispheric structures. To preserve RNA integrity, tissue blocks were submerged in RNAlater (Thermo Fisher) and stored at − 70 °C until processing. For histological analysis, an independent cohort of mice (n = 3 per treatment) underwent intracardiac perfusion with PBS followed by 4% PFA in PBS before whole-brain dissection. Tissue assignments for drug or vehicle treatment were randomized, and researchers were blinded to treatment identity until the completion of data analysis. All procedures were approved by the Animal Ethics Committee of Linköping University and conducted in accordance with ethical guidelines for animal research.

### Expression profiling

Total RNA was extracted using the RNeasy + Mini Kit (Qiagen) and processed with the QIAcube automated system (Qiagen). RNA integrity was assessed using Bioanalyzer RNA 6000 Nano Chips (Agilent), and all samples had an RNA integrity number (RIN) exceeding 8.5. RNA quantification was performed using Quant-iT (Invitrogen), and indexed sequencing libraries were constructed with the Illumina Stranded mRNA Prep Kit. Deep RNA sequencing was conducted using a NextSeq 2000 (Illumina, P50 flow cell) following the standard Illumina pipeline.

Paired-end reads were aligned to the mouse GRCm39 genome (June 2020) using HISAT2, with sequencing depths ranging between 20 and 40 million mapped reads per sample. Differential expression analysis was performed using DESeq2 within the R (R Core Team, 2023, www.r-project.org) and Bioconductor [14] framework, comparing gene expression in the injected hemisphere to that in the contralateral hemisphere. Additional comparisons were made between treatment groups, including R55 vs. vehicle, R33 vs. vehicle, and R55 vs. R33. Pathway and functional enrichment analyses were conducted using multiple databases, including Gene Ontology (GO), InterPro, KEGG, Monarch, Pfam, SMART, STRINGdb, UniProtKB, and WikiPathways.

To contextualize transcriptional alterations observed in 5xFAD mice, external reference datasets were used, including publicly available RNA-seq data from MODEL-AD datasets (GSE168137, [11]), which document transcriptomic changes in 5xFAD mice across disease progression, from Bliim, Leshchyns’ka, Keable, Chen, Curry-Hyde, Gray, Sytnyk and Janitz [5] (GSE110908), which describes gene expression changes following chemically induced long-term potentiation (cLTP) and [19] describing the disease-associated microglia (DAM) phenotypes in 5xFAD.

### Quantitative histology

Following transcardial perfusion, brains were cryoprotected in sucrose in 4% PFA until they lost buoyancy, then snap frozen. Sections were obtained using a cryostat and immunostained with the following primary antibodies: anti-VPS35 (clone 7E4, NBP2-78823, Novus Biologicals, CO), anti-RAB7 (clone E9O7, Cell Signaling, MA), anti-VPS13b (PA5-34406, Invitrogen), anti-SORL1 (NB100-785, Novus) and anti-IBA1 (ab5076, Abcam).

Fluorescence microscopy was performed using a Zeiss LSM700 confocal microscope, and image deconvolution was applied for signal enhancement. Cortical layer V neurons adjacent to the injection site, but sufficiently distant from the needle track to maintain normal parenchymal morphology, were segmented using QuPath [2]. Fluorescence intensity was quantified in approximately 700 segmented neurons per protein. The quantification was done in the cortex layer V, aiming to analyse as close to the injection track as possible but without artefacts from the procedure. For the Sorl1 study, the morphometry, location and distance to nuclear envelope of about 20,000 Sorl1 + particles was analysed using ImageJ DiAna plugin [12].

### Statistical analysis

All data were analyzed using R, with results presented as mean ± standard error (SE). For multiple comparisons, FDR corrected p-values are presented. The statistical significance threshold was set at α = 0.05. Two-tailed statistical tests were applied for comparisons.

## Results

### Selection of the 5xFAD model for retromer stabilization studies

**Fig. 1:**
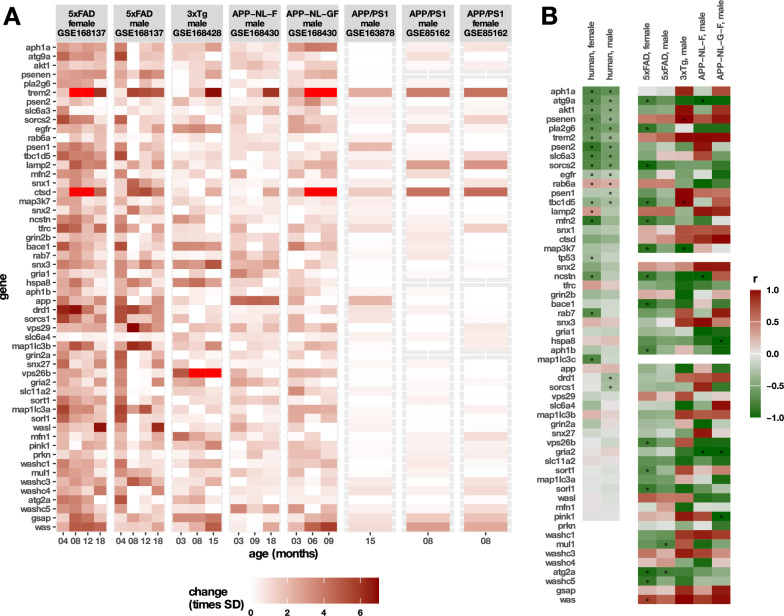
Temporal evolution of retromer interactome genes and cargo in transgenic AD models and human AD cohorts. **A** Temporal progression of retromer-related gene expression across multiple transgenic AD models. Expression changes are color-coded relative to the mean expression across transgenic and wild-type littermates at all ages and normalized to the standard deviation (SD). To enhance clarity, wild-type expression levels are not shown. **B** Correlation between expression of equivalent genes in human AD cohorts and disease progression, assessed by Mini-Mental State Examination (MMSE) scores (data from GSE185909). In transgenic mouse models, expression was analyzed as a function of age (data from the same GSE dataset as panel A). Correlations are presented separately by sex, with significant correlations (*p* < 0.05) indicated by"o"

To determine the most suitable mouse model for investigating retromer dysfunction in AD, we analysed temporal expression patterns of key genes within the retromer interactome and its cargo across multiple transgenic AD models, including APP NL-F knock-in, APP NL-G-F knock-in, APP/PS1, 3xTgAD, and 5xFAD (Fig. 1A). We compared these profiles to human AD datasets, assessing how gene expression evolved with disease progression and transgenic model age (Fig. 1B). Among these models, 5xFAD exhibited the most extensive and consistent changes, closely mirroring human AD pathology. Based on these findings, we selected 5xFAD mice as the primary model for this study.

### Retromer stabilization by R55 restores synaptic and neuroinflammatory pathways

**Fig. 2:**
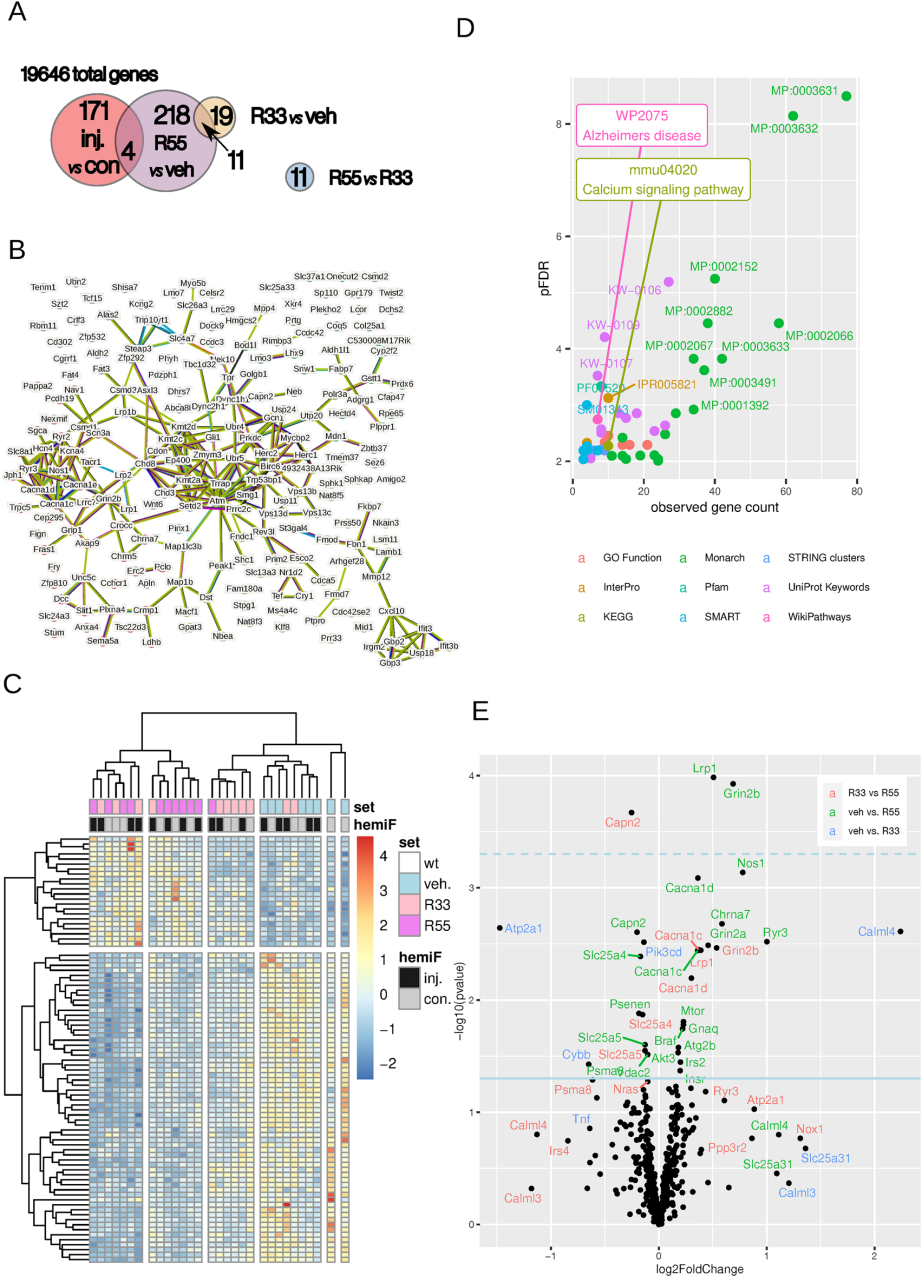
Differential gene expression and pathway enrichment following retromer complex stabilization by R55 and R33 in 5xFAD mice. **A** Summary of differentially expressed genes (DEGs) after treatment with R55, R33, or vehicle (Veh) in the injected (Inj) and contralateral (Con) hemispheres. Treatment and hemisphere were included as separate factors in the statistical model, and pairwise contrasts were conducted for each. **B** Interaction network of DEGs between R55 and vehicle, with significantly higher connectivity than expected by chance (*p* < 1 × 10^−16^). **C** Heatmap of significant DEGs between R55, R33 and vehicle across individual samples. **D** Pathway enrichment analysis of significantly altered genes in the R55 vs. vehicle comparison, color-coded according to the database source. Enrichment significance is represented as –log₁₀ (False Discovery Rate, FDR). All displayed terms are significant (*p* < 0.05). **E** Volcano plot illustrating log₂ fold changes of genes from the Alzheimer’s WP2045 dataset under different treatment conditions (R55, R33, and vehicle)

To assess the impact of retromer stabilization, 5xFAD mice received intracranial stereotaxic injections of R55 or R33, achieving an estimated final brain concentration of 25 µM. Vehicle-only injections served as controls. Twenty-four hours post-injection, brains were dissected for differential gene expression analysis. Hematoxylin and eosin staining of sections from the injection site showed the injection canal with intact surrounding tissue without signs of toxicity from the treatments. Neurons appeared with normal morphology after both vehicle, R55 and R33 injections (Supplementary Fig. 1), sections from R55 and R33 treated animals appeared to have a slight increase in microglia. Out of 19,646 detected genes, 218 (1.10%) were significantly differentially expressed in R55-treated mice compared to vehicle controls (Fig. 2A, GSE267989). R33 treatment resulted in 19 differentially expressed genes (0.10%), with most showing changes similar to R55 but of lower magnitude. Direct comparison of R55 vs. R33 revealed only 11 significantly altered genes (0.05%), supporting the hypothesis that R55 and R33 share a similar mechanism of action, with R55 exhibiting stronger effects at this time point. Thus, we focused mainly on R55 for the rest of the study, unless otherwise stated. Microinjection alone caused 171 genes (0.87%) to be differentially expressed between the injected and contralateral hemispheres. However, only four genes (*Cdca5, Esyt1, Ifi30, Mmp12*) overlapped with the R55 vs. vehicle dataset, suggesting minimal confounding effects from injection trauma (data not shown).

Network analysis of the R55-regulated genes identified 247 interactions with an average node degree of 2.38 (Fig. 2B), significantly exceeding the expected 101 interactions from a random dataset (*p* = 1.10e-16), demonstrating that R55 selectively modulates interconnected pathways. Hierarchical clustering revealed distinct gene expression signatures across hemispheres and treatment groups (Fig. 2C). Interestingly, gene expression changes were observed in both ipsilateral and contralateral hemispheres, suggesting widespread parenchymal distribution of the compounds.

Enrichment analysis using multiple databases, revealed that the majority of the enriched GO terms were neuronal and synaptic-related, as well as some related to calcium handling and axonal organization (Fig. 2D). The AD-related pathway WP2075 was significantly altered, along with synaptic organization pathways and other CNS-related phenotypes. Structural protein domain enrichment analysis highlighted common domains in the differential expression genes, specifically the ones related to PSD-95, *Egf*-like, and *Vps13* families (Fig. 2E). Notably, a volcano plot of WP2075 genes (Fig. 2F) confirmed that both R55 and R33 modulated gene expression in the same direction, with R55 exerting stronger effects.

### Retromer complex stabilization reverses synaptic deficits in 5xFAD mice

**Fig. 3:**
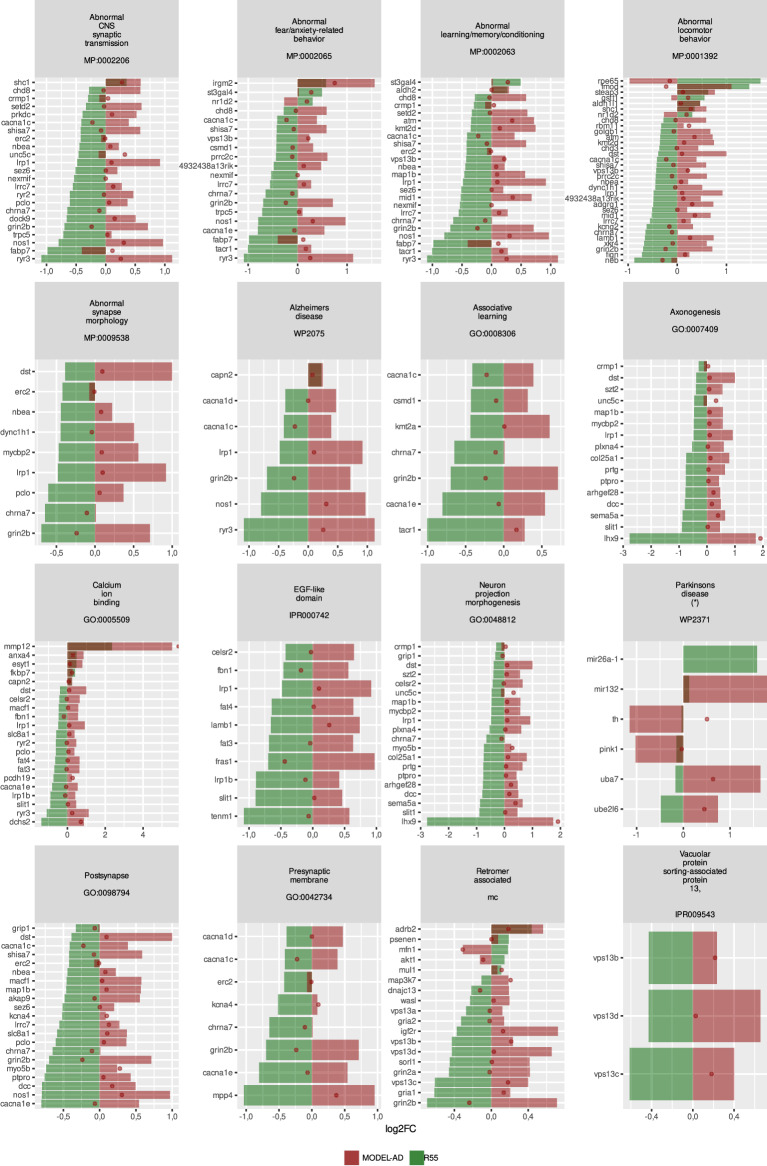
Comparative pathway analysis of retromer complex stabilization in 5xFAD mice relative to MODEL-AD datasets. Comparison of log₂ fold changes between R55-treated and vehicle-treated 5xFAD mice (green) and age-matched 5xFAD vs. wild-type (WT) controls (red) from the MODEL-AD dataset. For MODEL-AD data, dots represent 4-month-old mice, and solid bars represent 8-month-old mice. Gene sets were curated from multiple databases, avoiding redundancy and overly broad categories (e.g., abnormal nervous system phenotypes). Additional manually curated gene sets include retromer-associated genes, as no existing database comprehensively covered this group. Only genes significantly altered in the R55 vs. vehicle comparison are shown, except for WP2371 (Parkinson’s disease pathway), where all significantly altered genes from both comparisons are displayed

To evaluate the potential neuroprotective effects of retromer-directed therapy, we compared R55-induced gene expression changes with data from the MODEL-AD 5xFAD mouse reference dataset [11]. We selected relevant enrichment clusters and plotted gene expression changes at 4 months (dots) and 8 months (bars) of age in red, and overlaid R55-induced changes in green (Fig. 3). As none of the databases contain a defined cluster for retromer complex-related genes, we manually curated a set of retromer genes that also included relevant cargos to evaluate the effects in this cluster. For most genes, R55 treatment induced expression changes of similar magnitude but reversed to those observed at 8 months in 5xFAD mice, suggesting that retromer stabilization normalizes AD-related transcriptional alterations. This effect was largely specific to AD-related pathways, as genes from the Parkinson’s disease-associated WP2371 pathway showed minimal changes, with none reaching significance in the R55 vs. vehicle comparison.

**Fig. 4:**
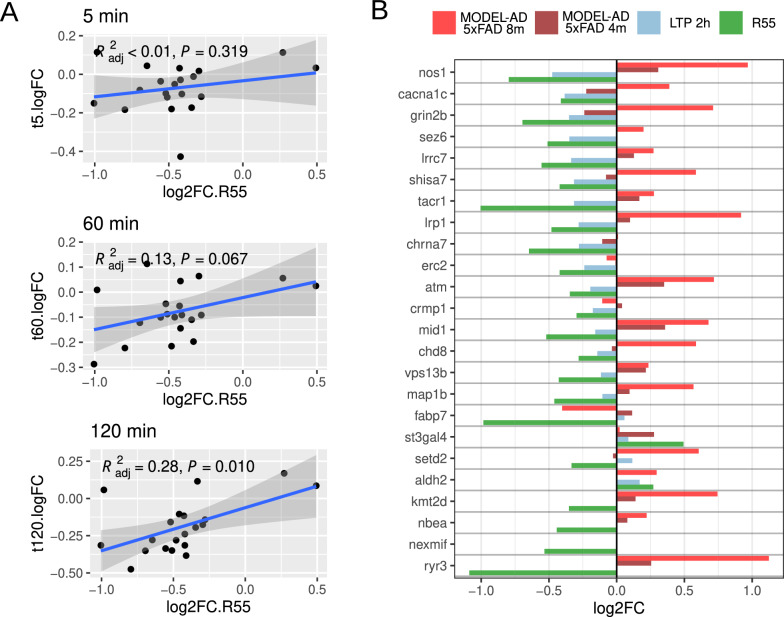
Retromer stabilization restores synaptic gene expression in 5xFAD mice. **A** Correlation of gene expression changes in genes related to abnormal learning, memory and conditioning in R55-treated 5xFAD mice (vs. vehicle) with those observed following chemically induced long-term potentiation (cLTP) at 5, 60, and 120 min, as reported by Bliim et al. [5]. **B** Expression changes of the same genes in our dataset, the Bliim et al. dataset, and the MODEL-AD dataset at 4 and 8 months, providing a temporal context for synaptic gene regulation in AD progression

To examine the effect on synaptic-related proteins in more detail, we investigated changes in the gene set corresponding to abnormal learning, memory, and conditioning phenotype (Monarch 2063). Comparing R55-induced changes to LTP time-course datasets [5], we found that R55-induced expression changes were strongly aligned with late-phase (2 h) LTP-associated transcriptional signatures (Fig. 4A). For most of these genes the expression changes induced by LTP is opposed to the AD pathological expression changes in the MODEL-AD dataset. Interestingly, the R55 treatment mostly resulted in gene expression changes in the same direction as late LTP, counteracting the 5xFAD changes (Fig. 4B). This suggests that R55 treatment counteracts the synaptic and LTP dysfunction observed in 5xFAD mice.

**Fig. 5:**
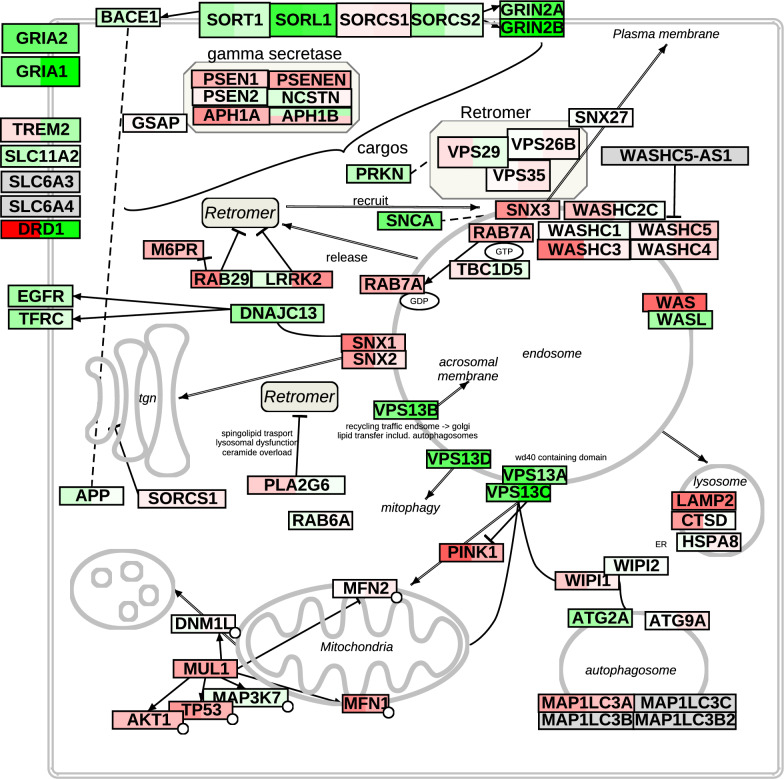
Summary of alterations in the retromer complex and related proteins relevant for Alzheimer's disease. This manually curated diagram summarizes transcriptional changes in retromer-associated genes and related proteins implicated in AD following treatment with R33 (left area in the box for each gene) and R55 (right area). Gene expression changes are represented using a color scale, where green indicates downregulation and red indicates upregulation, with color intensity proportional to the magnitude of change. Genes with minimal or statistically insignificant alterations are displayed in near-white for completeness. Unlike Figs. 3, 4, and 8, which show log₂ fold changes relative to vehicle controls, this figure presents mean Z-normalized values for each treatment in the injected hemisphere, similar to the heatmap in Fig. 2C

To summarize and visualize the changes caused by R55 and R33 treatment, we mapped differential expression onto a pathway diagram of endosomal processing in AD (Fig. 5). Interestingly, core retromer proteins (Vps35, Vps26a, Vps29) remained unchanged following R55 treatment and during disease progression (MODEL-AD), but their known cargos and associated proteins showed widespread alterations. This implies functional changes to the retromer that are not through expression changes, that has extensive effects on the endosomal system and many AD related cellular pathways. Notably, both R33 and R55 treatment resulted in consistent and significant changes in the entire Vps13 domain-containing family, which are critical for endosomal function.

### Retromer stabilization alters subcellular protein localization

**Fig. 6:**
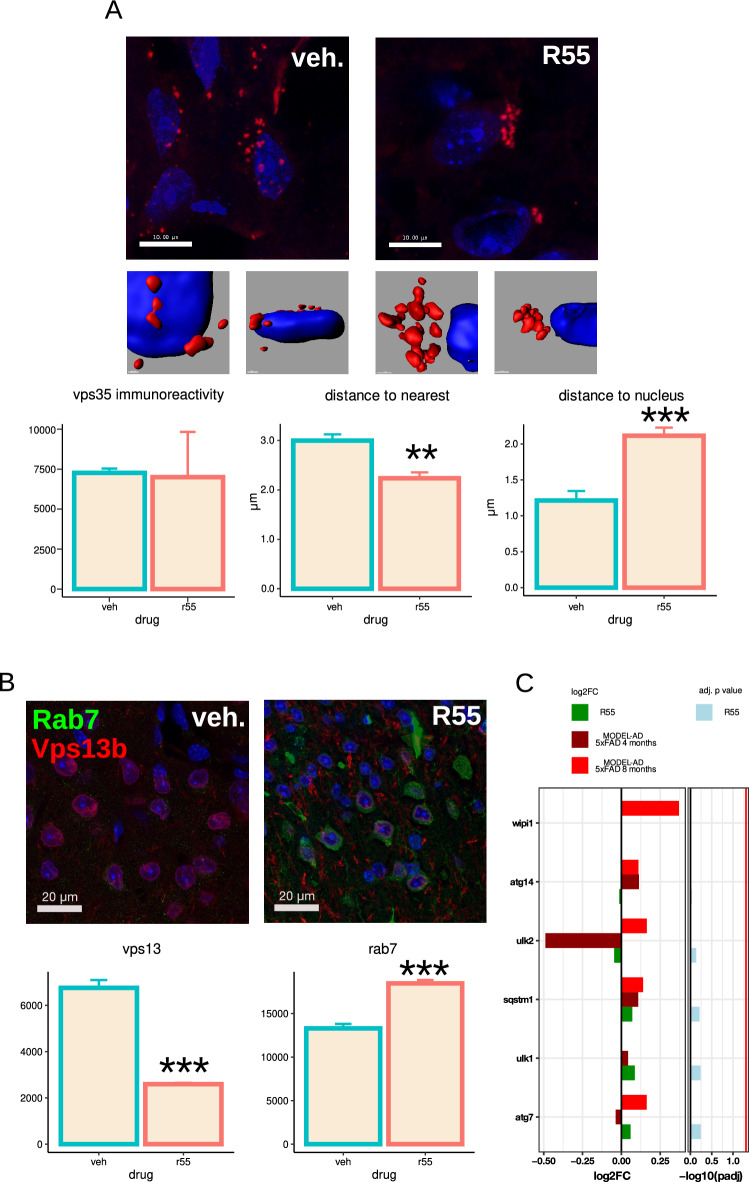
Immunohistochemical analysis of VPS35, VPS13, and RAB7 following R55 treatment in 5xFAD mice. **A** Representative images of VPS35 immunolabeling (red) in vehicle- and R55-treated mice (top). Nuclei labeled by DAPI. Mid panel shows the corresponding 3D reconstructions of the VPS35 immunolabeling. Quantification of the mean fluorescent intensity (arbitrary units) of VPS35-positive particles (bottom) shows no change in the amount of VPS35, but that R55 treatment reduce the distance to the nearest particle (increased VPS35 clustering) and the spatial distribution away from the nucleus. **B** Representative images of VPS13 (green) and RAB7 (red) immunolabeling in vehicle- and R55-treated mice (top). Quantification of VPS13 and RAB7 mean fluorescent intensity (arbitrary units) within segmented region of interest (bottom) confirms R55-induced VPS13 reduction and RAB7 upregulation. Note that background staining, most evident in the R55 image was not included in the segmentation which was done around each nucleus. **C** Expression changes of genes linked to the TFEB/TFE3 axis and lysosomal degradation in our dataset (green). No significant changes were observed in ATG7, ATG14, WIPI1, ULK1, ULK2, or SQSTM1 (red line shows *p* = 0.05), supporting the interpretation that lysosomal biogenesis was not enhanced after R55 treatment. MODEL-AD dataset at 4 and 8 months included for comparison

**Fig. 7:**
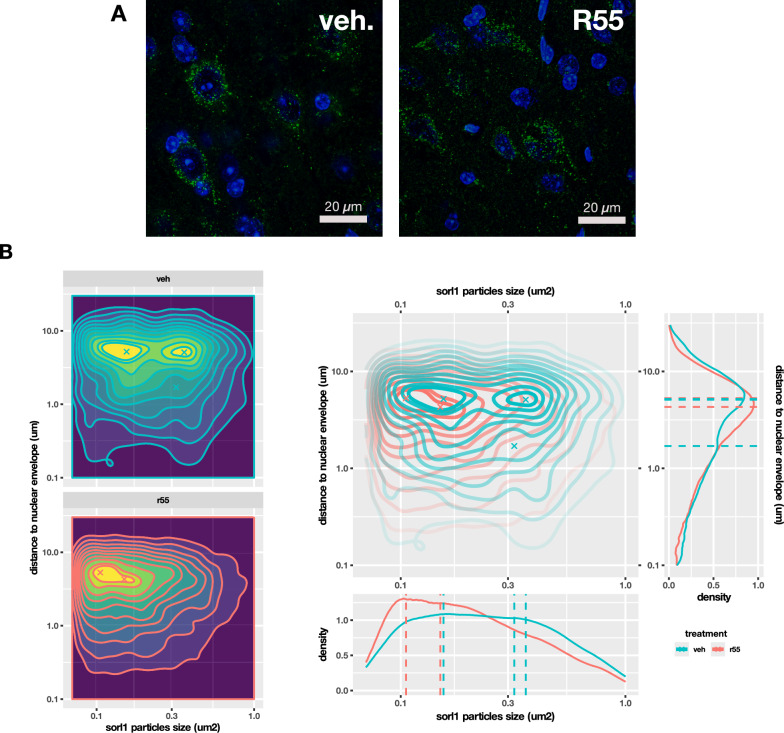
Retromer stabilization alters the size and cellular distribution of SORL1 labeled particles in a manner consistent with more efficient retromer function. **A** Representative Sorl-1-stained (green) images of R55-treated and vehicle-treated 5xFAD mice. **B** Density map of Sorl-1 particle size (horizontal axis) versus distance to the nuclear envelope (vertical axis). Increasingly warmer colors indicate increasing numbers of particles with each axis feature. In the vehicle-treated 5xFAD mice there is a significant number of larger particles that have travelled less (right yellow peak and shallow slope below), which is absent in the R55-treated mice. The curves represent the increase in population density from 0 to 90% in 10% steps, plus an additional step at 95%. The X's showing the local maxima are to aid interpretation of the next panel. **C** Overlay of the two 2D density maps and lateral projections on distance (vertical axis) and size (horizontal axis), color coded for vehicle and R55 treated 5xFAD mice. The Xs on the 2D maps are shown as dashed lines on the lateral 1D densities

Given the unexpected alterations in Vps13 gene family expression, we investigated whether retromer stabilization impacted subcellular protein distribution. As expected, VPS35 protein levels did not differ significantly between 5xFAD mice and WT litter mates (supplementary Fig. 2). Immunohistochemistry of VPS35, VPS13b, and RAB7 in the R55 microinjected cortex revealed a significant reduction in VPS13 protein levels, an increase in RAB7 and VPS35 protein levels remained unchanged (Fig. 6), all consistent with the gene expression results. In vehicle-treated 5xFAD mice, VPS35-positive vesicles were irregularly distributed, often near the nucleus. However, in R55-treated mice, VPS35-positive vesicles formed dense clusters and were significantly shifted away from the nucleus (Fig. 6), indicative of enhanced or restored endosomal maturation, potentially driven by normalized expression of VPS13 and RAB7. To determine whether the increase in Rab7 expression was accompanied by changes in lysosomal biogenesis, we examined the transcriptional expression of genes linked to the TFEB/TFE3 axis and lysosomal degradation [32]. No significant changes were observed in transcriptional markers of this pathway, such as ATG7, ATG14, WIPI1, ULK1, ULK2, or SQSTM1 [44], supporting the interpretation that lysosomal biogenesis was not enhanced after R55 treatment. To further examine the effect on retromer function we analysed the size and subcellular distribution of Sorl1 labelled particles after vehicle and R55 treatment (Fig. 7A) and plotted the data in population density maps (Fig. 7B). In 5XFAD vehicle-treated brains, Sorl1 particle size is distributed in a wide range between 0.1 and 0.3 μm^2^ with peaks at 0.15 and 0.36 μm^2^. When measuring the distance from the nuclear envelope, the main peak is at 5.2 μm, there is a second maximum at 1.7 μm consisting of larger particle sizes. Interestingly, in R55 treated brains this second peak is absent. Furthermore, in the vehicle-treated brains almost half of the particles (5th contour line on the overlap) are less than 1 μm from the nuclear envelope while in the R55-treated brains, only 20% of the particles (3rd contour line on the overlap) remain at this close position. In addition, the particle sizes are larger in the vehicle-treated brains. In summary, R55 treatment results in a normalization of Sorl1 particle sizes and distributions.

### Retromer stabilization modulates microglial activation

**Fig. 8:**
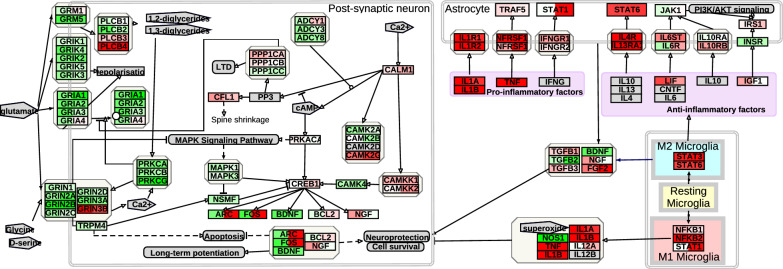
Retromer stabilization alters glutamatergic synapses, astrocyte function, and microglial activation. This diagram, adapted from WP5083, illustrates transcriptional changes in glutamatergic synapses, astrocytes, and microglia following treatment with R33 (left area in the box for each gene) and R55 (right area). Gene expression changes are represented using a color scale, where green indicates downregulation and red indicates upregulation, with color intensity proportional to the magnitude of change. Genes with minimal or statistically insignificant alterations are displayed in near-white for completeness. As in Fig. 5, this figure presents mean Z-normalized values for each treatment in the injected hemisphere, rather than log₂ fold changes relative to vehicle controls, as shown in Figs. 3, 4, and 8

As VPS35 plays a critical role in microglial activation [33], we investigated its impact on neuroglial interactions. Gene expression analysis after retromer stabilization revealed activation of microglial phenotype-associated genes, changes in astroglial receptors, inactivation of glutamatergic synapses and activation of some genes downstream of Creb in neurons. These changes caused by R55 and R33 treatment on neuroglial interactions with a focus on the glutamatergic synapse are summarized and visualized in an annotated pathway diagram (Fig. 8).

**Fig. 9:**
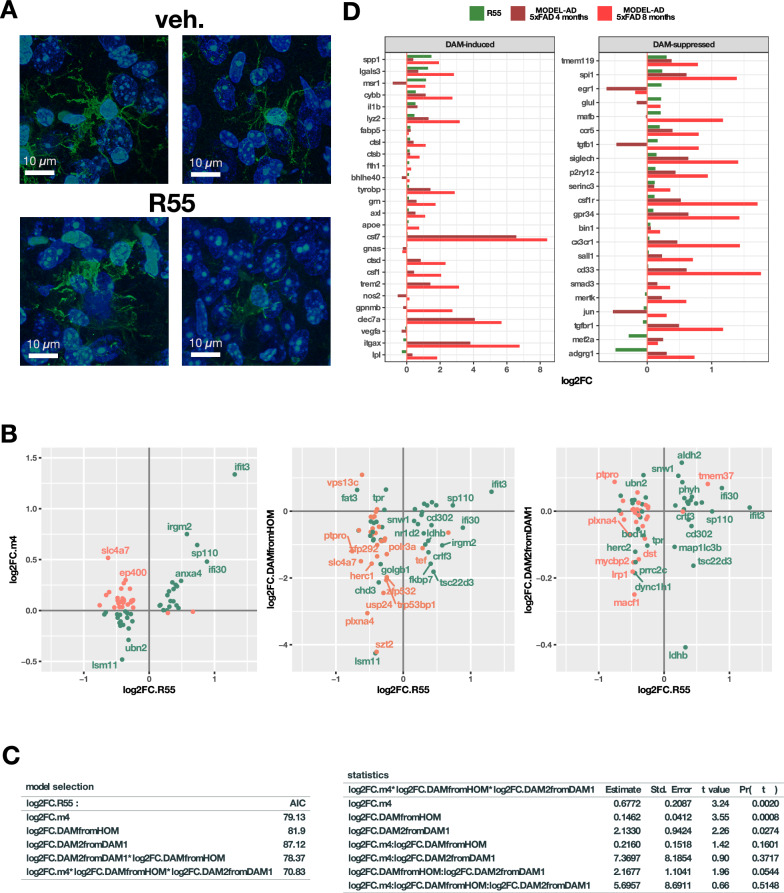
Retromer stabilization modulates microglial activation states in 5xFAD mice. **A** Representative IBA-1-stained (green) images of microglia in R55-treated and vehicle-treated 5xFAD mice. Nuclei labeled by DAPI. Microglia appear less ramified and complex following R55 treatment. **B** Comparison of the changes on drug treatment versus microglial activation. To localize the genes putatively responsible for the changes quantitatively observed in (**A**), we compared our changes in R55/vehicle-treated 5xFAD mice (log2FC R55) with differential expression on the microglial activation model described in [https://doi.org/10.1016/j.cell.2017.05.018], which includes differential expression from homeostatic microglia to DAM (logFC2 DAMfromHOM), differential expression from DAM I to DAM II (logFC2 DAM2fromDAM1). We also included the changes in MODEL-AD at 4 months (logFC2 m4). For this comparison, we only used genes with adjusted *p* < 0.05 in our treatment that were also present in the microglia study, so mostly microglia genes. Genes whose changes are of the same sign, both increased in R55 over vehicle and 5xFAD over WT, or both reduced, are shown in green. **C** Statistical modelling of (**B**). To statistically analyze the relevance of microglial activation in the changes observed after R55, we build a series of nested general linear models whose AIC (lower the better) relates to the goodness of fit. The model that includes both MODEL-AD and microglial activation has a better AIC and is significantly better than the others (likelihood ratio test vs. MODEL-AD alone: *p* = 0.003). We then calculated the contributions of each factor, including interactions, which can be observed in the right panel. The first three are significant. **D** Changes in microglial DAM-related gene expression following R55 treatment, contextualized against MODEL-AD datasets at 4 and 8 months

We further investigated the effect of retromer stabilization on microglia using IBA1 immunohistochemistry, this showed that microglia in R55-treated 5xFAD mice exhibited reduced ramification (Fig. 9A), suggesting further activation of microglia. To investigate these effects we compared the R55-induced differential expression changes with those reported by Keren-Shaul et al. [19] on FACS-sorted (CD45 +) immune cells from 5xFAD brains, characterizing the genes responsible for the homeostatic to DAM, and DAM I to DAM II phenotype. We intersect their microglia gene set with our R55 vs. vehicle data, and plotted the changes we observed against their changes, as well as the changes in MODEL-AD (Fig. 8B). The left panel compares our results in the genes from the Keren-Shaul dataset to MODEL-Ad at 4 months (Log2FC.R55 vs. Log2FC.m4). Genes that are compensated by R55 occupy the upper left and lower right quadrants and are coloured red. Genes with changes of the same sign in both models are coloured green. In the middle panel, R55 changes are plotted against the Keren-Shaul's changes from homeostatic microglia to DAM (log2FC.R55 vs logFC.DAMfromHOM). The colour coding of each gene is retained from the first diagram. This shows that a large majority of the originally"red"genes now change with the same sign, thus being consistent with microglial activation after R55 treatment. Next, we did the same against the changes from DAM I to DAM II2 (log2FC.R55 vs log2FC.DAM2fromDAM1), with similar results.

Finally, to obtain a statistical quantification, we build a series of generalized linear models of increasing complexity that express the changes we observed in R55/veh (log2FC.R55) as a function of the data in plot 8B separately (logFC.m4, log2FC.DAMfromHOM, log2FC.DAM2fromDAM1) or in combination. We calculated the Akaike information criterion (AIC) for each model and found that the model that included all factors had the best AIC (8C) and was statistically different from the others on the likelihood ratio test. We then estimated the statistical contribution of each parameter to the model and the combinations between them, and found that logFC.m4, log2FC.DAMfromHOM, log2FC.DAM2fromDAM1 contribute significant separately, and that log2FC.DAMfromHOM actually has a greater effect than log2FC.m4. The combination of microglial activation phenotypes (log2FC.DAMfromHOM * log2FC.DAM2fromDAM1) almost reaches statistical significance. Taking this all together, shows that the DAM from HOM and DAMII from DAM I changes explain a significant part of the changes we observe in microglial genes after R55 treatment (Figs. 9C, D).

## Discussion

### Retromer stabilization as a potential strategy to reverse AD-related molecular alterations

Disruptions in synaptic transmission and plasticity are early hallmarks of AD. Endosomal trafficking, mediated by the retromer complex, plays a pivotal role in synaptic function by regulating intracellular protein transport. Our study demonstrates that stabilizing the retromer complex reverses key molecular alterations in AD, shedding light on downstream mechanisms and signaling pathways affected by retromer dysfunction. Our results supports previous reports that dysfunctional retromer linked to neurodegenerative diseases is not primarily caused by loss of expression of Vps35 but by decreased endosomal association causing loss of function that can be mitigated by retromer stabilizing compounds such as R55 and R33 [10]. Additionally, our analysis confirms that 5xFAD mice exhibit extensive gene expression changes in the retromer interactome and its cargo, resembling patterns seen in human AD cohorts — a degree of alteration not observed in other transgenic AD models.

### Retromer stabilization restores synaptic plasticity and LTP-associated pathways

A strong body of evidence links synaptic dysfunction to early AD progression, with Aβ oligomers interfering and inducing changes similar to those involved in developmental synaptic pruning and with plasticity-related processes such as LTP and LTD [23]. Under normal conditions, these interactions are transient, but in AD, Aβ oligomers induce persistent synaptic alterations, contributing to disease progression. For example, Aβ oligomers increase cholesterol concentration at synapses, promoting Aβ/PrP complexes that disrupt NMDAR signaling via GRIN2a/GRIN2b S-nitrosylation [3]. In line with these events, we observed compensatory increases in Grin2a, Grin2b, iNos, and VLDL/ApoE-related genes such as *Lrp1*, *Lrp1b*, and *Fbn1* in 5xFAD mice (MODEL-AD datasets). Notably, retromer stabilization by R55 counteracted these transcriptional upregulations, suggesting enhanced receptor recycling and trafficking reduced the need for compensatory gene expression changes.

The retromer complex is crucial for synaptic AMPA receptor trafficking. Loss of VPS35 impairs Gria1 (AMPA) receptor insertion and disrupts LTP, while restoring VPS35 reverses these deficits [39]. In our study, R55 treatment normalized Gria1 and Grip1 expression, the latter being essential for AMPA receptor endosomal sorting and membrane targeting. This is particularly significant given recent findings implicating the semaphorin family (Plexin a4, Sema5a) and netrin receptors (Unc5c, Dcc) in AMPA receptor targeting during LTP-associated plasticity [17]. In 5xFAD mice, these genes were dysregulated, but their expression was restored by retromer stabilization, further supporting a role for retromer function in synaptic integrity.

### Retromer stabilization modulates calcium handling in AD pathology

Defective intracellular calcium homeostasis, particularly endoplasmic reticulum (ER) calcium leakage, is an early event in AD-linked synaptic dysfunction. The neuronal ryanodine receptor (Ryr2) is overactivated in AD, leading to enhanced receptor-operated calcium entry and decreased buffering capacity [18, 21]. Furthermore, RyRs interact functionally with L-type calcium channels (Cacna1c, Cacna1d, Cacna1e), particularly in CA1 pyramidal neurons, where calcium-induced calcium release contributes to synaptic plasticity [35].

In 5xFAD mice, we observed elevated Ryr2 and Cacna1c/Cacna1d/Cacna1e expression, consistent with previous findings. R55 treatment effectively reversed these alterations, suggesting restored calcium signalling homeostasis. Of Ryr2-associated regulators [38], only Jph4 showed a weak response to R55, indicating selective rather than global modulation of calcium-handling genes.

### Effects of retromer stabilization on Aβ processing and endosomal function

Given that the retromer complex regulates Aβ processing, we examined key AD-related cargos. R55 treatment increased γ-secretase components while reducing Sorl1 and Col25a1 expression. *Col25a1* encodes a collagen-like amyloid plaque component, known for forming protease-resistant aggregates. These changes suggest that recovered retromer function may reduce excessive amyloidogenic processing caused by extended cargo residency at the Golgi by normalizing Golgi-retromer dynamics.

Unexpectedly, R55 treatment significantly reduced Vps13 family gene expression, which was otherwise elevated in 5xFAD mice. The Vps13 complex regulates endosomal recycling towards the Golgi, mitochondria, and autophagosomes by promoting organelle bilayer bridging, and has been implicated in mitochondrial dysfunction in neurodegenerative diseases, including atypical PARK23 [40]. Genetic variations in VPS13 genes are emerging risk factors for sporadic, late-onset AD, with VPS13 variants exceeding even ApoE in risk contribution [27]. Immunohistochemistry confirmed that Vps13 protein levels decreased following R55 treatment, while RAB7 expression increased, this suggests restored endosomal maturation. Previous studies have shown a stagnation of large endosomes both in AD and in 5xFAD [9, 24] and impaired recycling pathway, in 5xFAD in mice of almost the same age [24]. We used SORL1 expression as a marker of endosomal trafficking. SORL1 expression has been shown to have a reciprocal role prototypical cargos such as transferrin to in cell surface recycling [30]. Sorl1 distribution that has been reported to be altered both in APP overexpressing AD models such as 5xFAD and in Sorl1 mutations that have been linked to AD pathology [29]. The changes to Vps13 and RAB7, taken together with the increased proximity of both VPS35 and SORL1 to the plasma membrane, compensation in synaptic-related pathways, and the absence of changes in lysosomal degradation-associated genes, our data support improved sorting and membrane trafficking that may favor recycling over degradation.

### Retromer stabilization modulates neuroinflammation and microglial polarization

In the neuroinflammatory domain, R55 treatment increased both pro-inflammatory mediators (Il1, Nfkb2) and pro-trophic glial markers (Il4r, Il13ra1), as well as downstream anti-inflammatory transcription factors (Stat6, Stat3). This coordinated shift in crosstalk between microglial polarization and astrocytic responses mirrors findings reported in other AD studies [45]. Similarly, *Vps35* KO impaired microglial activation in response to Aβ [33].

IBA1 staining revealed that R55-treated 5xFAD microglia exhibited reduced ramification, indicating an altered activation state. Further gene expression comparisons to MODEL-AD and DAM datasets showed significant shifts in microglial phenotypes [8, 15]. Given that phagocytic receptors and cytokine receptors utilize retromer-mediated recycling in glia, these findings provide a mechanistic link between retromer function and neuroinflammation although further studies are needed to assess if the identified changes have long term positive or negative effects.

### Limitations of the study

This study focuses on dysregulation of the transcriptome in early AD and the acute molecular effects of retromer stabilizing drugs. Although our results suggests that retromer dysfunction can contribute to many AD related alterations and that the retromer stabilizing drugs have the potential to counteract these effects, further studies are needed to confirm these findings. Comparing the changes we identify to downstream effects on the proteome would be interesting, but large-scale proteomics are still challenging and currently there are no database for changes to the proteome similar to the MODEL-AD. As the focus of this study was on acute changes, we can only speculate on the long-term effects on AD-related phenotypes such as memory, cognition, and synaptic plasticity. Further studies assessing pharmacokinetics and pharmacodynamics of this type of drugs are also needed.

### Implications and future directions

While our study provides acute mechanistic insights, extrapolating these findings to chronic AD progression remains challenging. However, our results strongly support retromer dysfunction as a key contributor to synaptic pathology in AD, raising the possibility that retromer stabilization could mitigate disease progression.

One intriguing question is why VPS35 mutations (e.g., D620N) cause Parkinson’s disease (PARK17) rather than AD. The D620N mutation disrupts VPS35 binding to WASH complex proteins, affecting actin nucleation, while AD-linked VPS35 mutations (e.g., L625P) impair VPS35-VPS26-VPS29 assembly [25, 34]. Notably, R55 enhances VPS35 assembly without affecting WASH binding [4]. However, R55-treated 5xFAD mice exhibited changes in WASH complex components, sorting nexins (Snx1, Snx2, Snx3), RAB7, and Vps13, suggesting a broader role for retromer stabilization in intracellular trafficking beyond cargo selection.

Our findings suggest that retromer stabilization influences not only endosomal cargo selection but also endosomal anchoring to other organelles. The observed redistribution of VPS35-positive vesicles away from the nucleus suggests that endosomes are repositioned towards the plasma membrane, likely improving membrane protein recycling, the decrease in Vps13 and increase in RAB7 further support this hypothesis.

## Conclusion

We have identified a range of retromer-regulated cargos, including membrane receptors and synaptic proteins, that are dysregulated in early AD and restored by retromer stabilization. Our data suggest that retromer dysfunction contributes to synaptic and neuroinflammatory alterations in AD, and that stabilizing the retromer complex enhances endosomal recycling, synaptic receptor trafficking, and microglial activation states. As 5xFAD mice lack genetic alterations in VPS35 or other retromer components, our findings may be generalizable to sporadic AD cases, supporting the exploration of retromer-directed therapies as a potential disease-modifying strategy.

## Supplementary Information

Supplementary Materials

## Data Availability

The datasets generated and analyzed during the current study are available in in GEO with the accession GSE267989 (https://identifiers.org/geo:GSE267989). The MODEL-AD dataset analyzed during the current study is available in GEO with the accession ID GSE168137 (https://identifiers.org/geo:GSE168137). The cLTP dataset analyzed during the current study is available in GEO with the accession ID GSE110908 (https://identifiers.org/geo: GSE110908). The raw images and histological data from the current study available from the corresponding authors on reasonable request.
