# Supplementary material for: Stabilizing the retromer complex rescues synaptic dysfunction and endosomal trafficking deficits in an Alzheimer’s disease mouse model
Source: Acta Neuropathol Commun. 2025 Sep 10;13:190. doi: 10.1186/s40478-025-02096-8 (PMC12424221; doi:10.1186/s40478-025-02096-8)
Supplement: Supplementary file 1 — Supplementary Materials [file 40478_2025_2096_MOESM1_ESM.pdf]

Title

Stabilizing the Retromer Complex Rescues Synaptic Dysfunction and Endosomal Trafficking Deficits in an Alzheimer's Disease Mouse Model

Authors:

David Ramonet (1), Anna Daerr (1), Martin Hallbeck (1)

(1) Department of Biomedical and Clinical Sciences and Department of Clinical Pathology, Linköping University, 581 85, Linköping, Sweden.

Corresponding authors: David Ramonet and Martin Hallbeck

David.Ramonet@liu.se

Anna.Daerr@liu.se

Martin.Hallbeck@liu.se

Supplementary figures

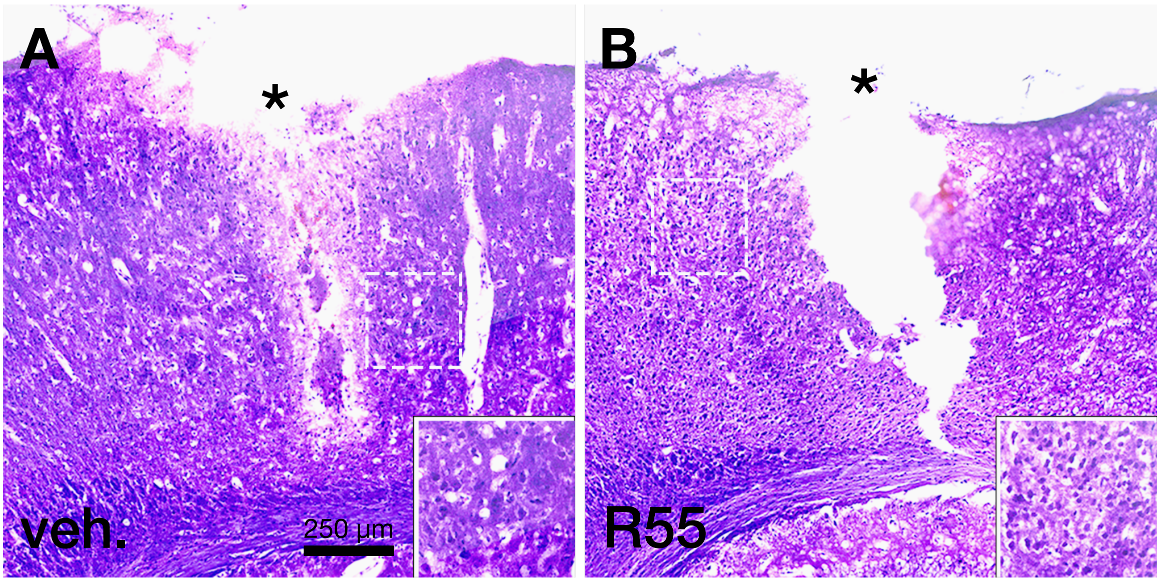

Supplementary figure 1 Injection of R55 does not cause toxic damage to the brain tissue. Hematoxylin and eosin staining of sections from the injection site of vehicle (A) or (B) R55 showing the injection canal with intact surrounding tissue without signs of toxicity from the treatments. Inserts showing magnification. Neurons appear with normal morphology after both vehicle and R55 injections, sections from R55 treated animals appeared to have a slight increase in microglia.

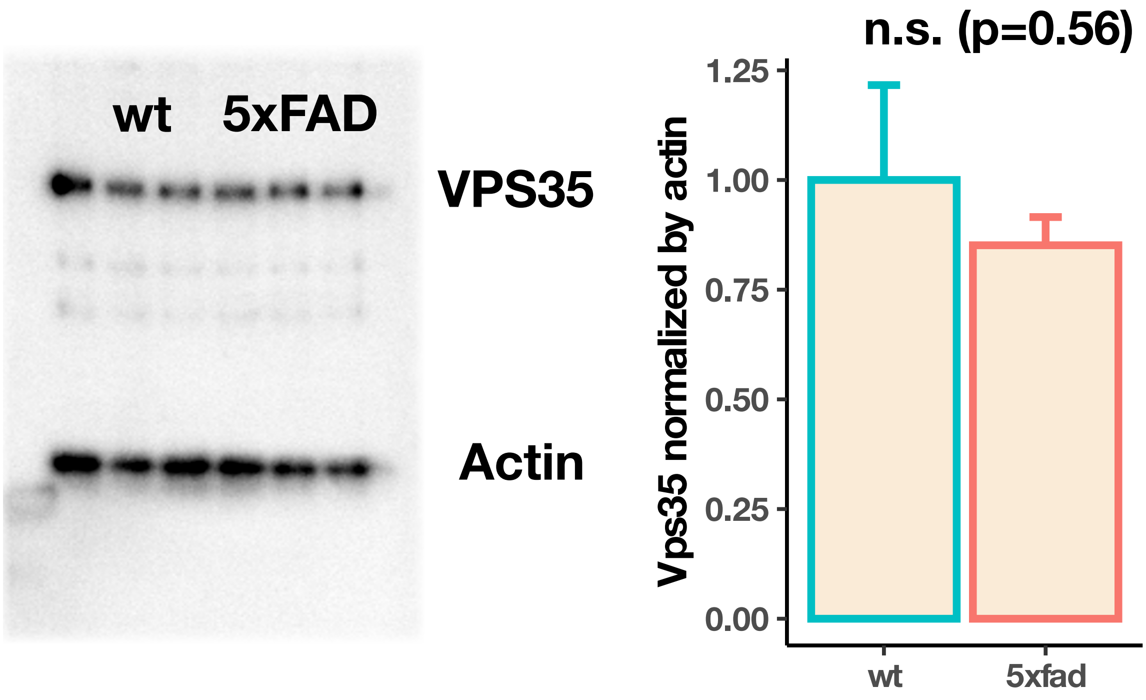

Supplementary figure 2 We obtained protein extracts from 5xFAD and WT at 4 months of age, prepared a protein homogenate and blotted against Vps35 antibody and actin. Quantification showed no changes between them.
